# Childhood ADHD and Risk for Substance Dependence in Adulthood: A Longitudinal, Population-Based Study

**DOI:** 10.1371/journal.pone.0105640

**Published:** 2014-08-27

**Authors:** Sharon Levy, Slavica K. Katusic, Robert C. Colligan, Amy L. Weaver, Jill M. Killian, Robert G. Voigt, William J. Barbaresi

**Affiliations:** 1 Division of Developmental Medicine, Boston Children’s Hospital, Harvard Medical School, Boston, Massachusetts, United States of America; 2 Department of Health Sciences Research, Mayo Clinic, Rochester, Minnesota, United States of America; 3 Department of Psychiatry and Psychology, Mayo Clinic, Rochester, Minnesota, United States of America; 4 Department of Pediatrics, Texas Children’s Hospital, Baylor College of Medicine, Houston, Texas, United States of America; Alexander Fleming Biomedical Sciences Research Center, Greece

## Abstract

**Background:**

Adolescents with attention-deficit/hyperactivity disorder (ADHD) are known to be at significantly greater risk for the development of substance use disorders (SUD) compared to peers. Impulsivity, which could lead to higher levels of drug use, is a known symptom of ADHD and likely accounts, in part, for this relationship. Other factors, such as a biologically increased susceptibility to substance dependence (addiction), may also play a role.

**Objective:**

This report further examines the relationships between childhood ADHD, adolescent- onset SUD, and substance *abuse* and substance *dependence* in adulthood.

**Method:**

Individuals with childhood ADHD and non-ADHD controls from the same population-based birth cohort were invited to participate in a prospective outcome study. Participants completed a structured neuropsychiatric interview with modules for SUD and a psychosocial questionnaire. Information on adolescent SUD was obtained retrospectively, in a previous study, from medical and school records. Associations were summarized using odds ratios (OR) and 95% CIs estimated from logistic regression models adjusted for age and gender.

**Results:**

A total of 232 ADHD cases and 335 non-ADHD controls participated (mean age, 27.0 and 28.6 years, respectively). ADHD cases were more likely than controls to have a SUD diagnosed in adolescence and were more likely to have alcohol (adjusted OR 14.38, 95% CI 1.49–138.88) and drug (adjusted OR 3.48, 95% CI 1.38–8.79) dependence in adulthood. The subgroup of participating ADHD cases who did not have SUD during adolescence were no more likely than controls to develop new onset alcohol dependence as adults, although they were significantly more likely to develop new onset drug dependence.

**Conclusions:**

Our study found preliminary evidence that adults with childhood ADHD are more susceptible than peers to developing *drug dependence*, a disorder associated with neurological changes in the brain. The relationship between ADHD and alcohol dependence appears to be more complex.

## Introduction

Individuals with attention-deficit/hyperactivity disorder (ADHD) are known to be at significantly greater risk for the development of substance use disorders (SUD) compared to their non-ADHD peers. [1]–[3] The *Diagnostic and Statistical Manual of Mental Disorders, Fourth Edition-Text Revision* (DSM-IV-TR) had defined two types of SUDs: *substance abuse* (substance use that interferes with daily functioning or is associated with recurrent problems) and *substance dependence* (manifested by an intense desire for the drug of choice and loss of control over use). [4] (The nomenclature was changed to mild-, moderate- or severe- SUD in the Diagnostic and Statistical Manual of Mental Disorders, Fifth Edition (DSM-5), [5] with “substance abuse” roughly correlating to moderate SUD and “substance dependence” to severe SUD. In this manuscript we will use the terms “abuse” and “dependence” because we are reporting on data from a standardized neuropsychiatric interview that was validated based on DSM-IV terms and definitions.) Research over the past decade has revealed that substance dependence (addiction, or severe SUD) is a neurologically-based disorder resulting from disruption of neurons in the reward center of the brain. This disruption results from repeated exposure to a psychoactive substance. [6]–[8] While substance *abuse* and substance *dependence* are grouped together as “substance use disorders”, they are clearly different: substance *dependence* involves a distinct pattern of neurological changes caused by repeated exposure to a psychoactive substance, while substance *abuse* does not.

Human brain development occurs in an orderly, rostral-caudal progression. Notably, the nucleus accumbens, sometimes referred to as the “reward center,” is mature by mid-adolescence, while the maturation of the pre-frontal cortex continues until the mid-20′s. [9] A body of epidemiologic data and analysis has established that the earlier an individual initiates psychoactive substance use, the more likely she or he is to ultimately develop substance dependence, with a near linear relationship. [10]–[13] In the population at large, by early adulthood, individuals seem to develop a relative resistance to substance dependence and the incidence falls dramatically. The declining incidence is thought to be related in part to maturation of the pre-frontal cortex [14], [15] and attendant maturation of “executive functions.”

Previously, we reported on the association between ADHD and SUD prior to 18 years of age among research-identified childhood ADHD cases and controls from a population-based birth cohort. [1] Our childhood ADHD cases were 6.2 times more likely (95% CI = 4.0–9.4; p<0.001) to have an alcohol/drug use disorder documented in their medical and/or school records than non-ADHD controls from the same birth cohort. Other investigators have also clearly demonstrated this relationship. [16]–[18] In our previous study, the association between ADHD case status and SUD was consistent for both boys and girls. Stimulant treatment tended to be protective, especially in boys, with significantly fewer stimulant-treated boys having a documented SUD during adolescence compared to boys with ADHD who were not treated with stimulants. [1].

The mechanisms underlying the relationship between ADHD and SUD are not fully understood, and previous research has not distinguished between substance *abuse* and substance *dependence* among individuals with ADHD. This distinction, however, may help to elucidate the complex relationship between the two disorders. This report examines the relationships between ADHD, substance abuse, and substance dependence among adults with research-identified childhood ADHD versus controls in order to explore whether individuals with ADHD are more susceptible to developing the neurological disorder of substance dependence in adulthood, after the typical period of heightened vulnerability in adolescence.

## Methods

### Study Setting

Rochester, Minnesota is geographically isolated in southeastern Minnesota and virtually all medical care for residents of Rochester is provided by Mayo Clinic, Olmsted Medical Center, and their 3 affiliated hospitals. The resources of the Rochester Epidemiology Project (REP) provide infrastructure for population-based research. [19], [20] All medical diagnoses and surgical procedures are recorded and indexed for computerized retrieval. For this ADHD study, all 41 public and private schools in Minnesota Independent School District 535 (city of Rochester school system) participated in a contractual research agreement that gave us permission to access their cumulative educational records for every child from the 1976–1982 Rochester, MN birth cohort. Written informed consent was obtained from all participants in the prospective portion of this study. The study was approved by the Institutional Review Boards of Mayo Clinic and Olmsted Medical Center.

### Retrospective Phase of the Study

#### Birth Cohort

This study employed a birth cohort consisting of all children born between January 1, 1976, and December 31, 1982, to mothers residing in the townships comprising Minnesota Independent School District 535, who continued to live in Rochester until at least age 5 years (N = 5718). Nineteen subjects with severe intellectual disability were subsequently excluded.

#### Identification of Childhood ADHD Cases and Controls

Our approach to identify ADHD incidence cases consisted of several steps, used multiple sources of information, and relied on recorded history of symptoms, individual test results (psychological assessments, including ADHD rating scales), and treatment documented in medical and school records that were available for all members of the birth cohort. Details of this process were previously reported. [21] Briefly, several steps were used to narrow the pool of potential ADHD incident cases, starting with cumulative school records of each child in the birth cohort (n = 5718). School records were searched for indications of concern about learning and/or behavior; 1961 children were identified with such concerns documented by teachers, parents, school psychologists, physicians, social workers, or/and school nurses. Next, we abstracted all descriptions of ADHD symptoms consistent with DSM-IV, results from teacher/parent ADHD questionnaires and clinical diagnoses of ADHD from school and medical records.

Identification of ADHD incident cases consisted of applying research criteria to these 1961 children. Subjects were defined as research-identified ADHD incident cases if their school/medical records included various combinations of three different categories of information: 1) met DSM-IV criteria for ADHD, 2) positive ADHD questionnaire results, 3) clinical diagnosis of ADHD. A total of 379 childhood ADHD incident cases were identified. Research diagnostic criteria were met at a mean age of 10.4 years. Non-ADHD controls were identified from the remaining members of the birth cohort who were in the community after 5 years of age, and who had *not* fulfilled research criteria for childhood ADHD.

#### Identification of Childhood Psychiatric Disorders among ADHD Cases and Controls

Details regarding the identification of co-occurring psychiatric disorders in childhood among ADHD cases and controls in this birth cohort are described elsewhere, [21] and consisted of a systematic, multistage process, utilizing detailed, routinely collected data as part of the Rochester Epidemiology Project. [19] This process included systematic abstraction of data from the medical records of ADHD cases and controls. To be classified with a psychiatric disorder in this study, we required documentation of (a) the initial diagnosis and (b) separate confirmation of the initial diagnosis prior to 18 years of age.

#### Identification of Adolescent Onset SUD’s among ADHD Cases and Controls

Information about alcohol and/or drug abuse was available in school records (e.g., school psychologist notes, guidance counselor reports, summaries of school assessments, social workers’ notes) and/or medical records (social worker, psychology and psychiatry records including correspondence regarding treatment for substance abuse provided by private treatment facilities). The school and medical records of each of the ADHD case and control subjects were reviewed by trained abstractors using a detailed protocol, for documentation of a clinical diagnosis of SUD. Additionally, the computerized Medical Diagnostic Index was also searched for any diagnosis related to or including “substance use disorder” among the 379 childhood ADHD cases under study and their matched controls. Adolescent onset “substance use disorder” required documentation of SUD in the medical and or school records prior to 18 years of age. [1].

#### Stimulant Medication Treatment

Medical and school records for every member of the birth cohort were reviewed and all documentation about treatment with stimulant medications was abstracted. We have previously described stimulant treatment among this cohort of ADHD cases, including age of onset, dosage, and duration of treatment throughout childhood. [22].

### Prospective Phase of the Study

#### Recruitment for Prospective Study

Of the 379 subjects with research-identified childhood ADHD identified in our retrospective study, 362 provided continued permission to access their medical records for research and were invited to participate in the adult outcome study. To ensure enrollment of sufficient non-ADHD controls, a random sample of 801 adults from the same birth cohort was also invited to participate. Five control subjects in the prospective study stated they had received a diagnosis of childhood ADHD. We reviewed records from these 5 subjects and determined that they had fulfilled our research criteria for childhood ADHD, but had not been thus classified previously because they had moved from the community before receiving this diagnosis or because of the timing of the original data abstraction. These 5 subjects were therefore reclassified as childhood ADHD cases for all analyses presented in this paper.

#### Identification of Adult Psychiatric Disorders and SUDs among Childhood ADHD Cases and Controls

All participants in the prospective study were administered the Mini International Neuropsychiatric Interview (M.I.N.I), including the module for Adult ADHD. [23], [24] The M.I.N.I. is a structured diagnostic interview for DSM-IV-TR and ICD-10 psychiatric disorders. Psychiatric disorders were determined by symptoms consistent with DSM-IV TR/ICD 10 as specified by the M.I.N.I.

The M.I.N.I. includes modules on “Alcohol Abuse and Dependence”. Each subject was first asked, “In the past 12 months, have you had 3 or more alcoholic drinks within a 3 hour period on 3 or more occasions?” All subjects responding “yes” to this first item were then asked a series of questions to determine classification as *current* alcohol dependence or *current* alcohol abuse. For the purposes of this study, subjects who reported consuming 3 alcoholic drinks within a 3 hour period on 3 or more occasions, but who did not meet criteria for alcohol abuse or dependence, were defined as having *heavy alcohol use.* Alcohol *abuse* and alcohol *dependence* were defined as per the M.I.N.I. Because the M.I.N.I does not ask about lower levels of alcohol use, subjects who did not report “heavy drinking” were defined as *no heavy alcohol use*. The M.I.N.I. also includes modules for “Non-Alcohol Psychoactive Substance Use Disorders.” The latter modules are completed for each psychoactive substance used in the past 12 months to “get high, to feel better, or to change your mood.” According to the M.I.N.I. administration protocol, a diagnosis of “dependence” pre-empts a diagnosis of “abuse” for all substances. For the purposes of this study, we defined any illicit substance use that did not meet criteria for drug abuse or dependence as drug *use without a disorder*. Drug *abuse* and drug *dependence* were defined as per the M.I.N.I., and drug *exposure* was defined as drug *use*, drug *abuse,* or drug *dependence*.

#### SUD Treatment

All subjects completed an extensive psychosocial questionnaire that included specific questions about having participated in a treatment program for “alcohol or drinking problems”, “marijuana use,” or “other street drugs or hard drugs.”

#### Statistical Methods

Characteristics were compared between participating and non-participating ADHD cases and between participating ADHD cases and participating non-ADHD controls using the two-sample t-test for age, the Fisher’s exact test for race, and the chi-square test for all other categorical variables. Current SUD outcomes in young adulthood (as determined from the M.I.N.I.) were first compared overall between individuals with childhood ADHD and non-ADHD controls and then separately stratified by adolescent onset SUD. The association between the 4-level current drug exposure status (no use, drug use without a disorder, abuse, dependence) and ADHD case status was assessed based on fitting a generalized logits model. The association between current drug dependence and ADHD case status was assessed based on fitting a binary logistic regression model. All logistic regression models included gender and age at participation and all reported odds ratios (OR) are reported as ORs adjusted for age and gender unless otherwise noted. The same approach was used to analyze current alcohol exposure. The Sobel test was used to test whether the estimate of mediation for co-occurring childhood psychiatric diagnoses was statistically significant. [25] All calculated p-values were two-sided and p-values less than 0.05 were considered statistically significant. Analyses were performed using SAS version 9.2 software (SAS Institute, Inc.; Cary, NC; 2010).

## Results

### Description of Participants in the Prospective Phase of the Study

Participating adults with childhood ADHD (n = 232) were similar to non-participants (n = 152) in regard to gender, age, ethnicity, rates of co-occurring mental health disorders, and stimulant treatment history. Control subjects were slightly older at the time of follow-up participation, more likely to be female, and significantly less likely to have had a co-occurring mental health disorder during childhood as compared to ADHD cases (Table 1).

**Table 1:** Comparison of demographic factors, co-occurring mental health disorders and stimulant treatment among participating ADHD cases, non-participating ADHD cases and controls.

Characteristic		ADHD cases		Participating Non-ADHD Controls(N = 335)	Participating ADHDcases vs. Non-participatingADHD cases,	Participating ADHD casesvs. ParticipatingNon-ADHD controls, *P*
		ParticipatingADHD cases(N = 232)	Non-ParticipatingADHD cases(N = 152)			
Male, n (%)		167 (72.0)	121 (79.6)	210 (62.7)	0.09	0.02
Age at time of participation (years)	Mean (SD)	27.0 (2.6)	–	28.6 (2.2)	–	<0.001
	Range	21.7–33.5	–	24.3–33.8	–	
White, n (%)		231 (99.6)	150 (98.7)	329 (98.2)	0.57	0.25
Received stimulanttreatment, n (%)		178 (76.7)	119 (78.3)	–	0.72	–
Psychiatric diagnosisprior to age 18,n (%)	Unknown, n	0	17†	0	0.92	<0.001
	No	101 (43.5)	58 (43.0)	281 (83.9)		
	Yes	131 (56.5)	77 (57.0)	54 (16.1)		
Substance use disorder(SUD) prior to age 18,n (%)	Unknown, n	0	17†	0	0.75	<0.001
	No	177 (76.3)	105 (77.8)	320 (95.5)		
	Yes	55 (23.7)	30 (22.2)	15 (4.5)		

† Seventeen of the non-participating ADHD cases had not authorized access to their medical records for research purposes at the time of the abstraction of these two parameters.

### Substance Use Disorders in Adolescence and Adulthood Among ADHD Cases and Controls

ADHD cases were significantly more likely to have SUD diagnosed during adolescence compared to controls (23.7% vs. 4.5%; unadjusted OR 6.63, 95% CI 3.64–12.08; Table 1). Among the 55 ADHD cases with adolescent onset SUD, the median age of SUD documented in the medical and school records was 16.3 years (range 13.2–18.0), while among the 15 controls with adolescent onset SUD, median age of SUD was 16.9 years (range 15.4–17.8). ADHD cases were also more likely to meet criteria for drug dependence (10.8% vs. 2.7%; adjusted OR 4.42, 95% CI 1.95–9.99, p<0.001) and alcohol dependence (14.7% vs. 8.7%; adjusted OR 1.87, 95% CI 1.07–3.27, p = 0.03) in adulthood. Marijuana was the most common substance of dependence among participating cases and controls (Table 2).

**Table 2:** Subjects with drug dependence in adulthood, by substance†.

Substance	ADHD Cases N = 232	Controls N = 335
Marijuana	16	6
Cocaine	11	4
Stimulants	10	2
Narcotics‡	3	1
Tranquilizers*	3	0
Hallucinogens††	2	0

† Some subjects were found to be dependent on more than one substance. A total of 18 ADHD cases and 5 controls were dependent on substances other than marijuana.

‡ Includes heroin, morphine, Dilaudid, opium, Demerol, methadone, codeine, Percodan, Darvon, OxyContin.

*Includes quaalude, Seconal (“reds”), Valium, Xanax, Librium, Ativan, Dalmane, Halcion, barbituates, Miltown.

†† Includes LSD (“acid”), mescaline, peyote, PCP (“Angel dust”, “peace pill”), psilocybin, STP, “mushrooms”, ecstasy, MDA, MDMA.

### Drug Use in Adulthood by Adolescent Onset SUD Status

Among the subgroup of participants who did not have adolescent onset SUD, rates of drug exposure in adulthood (i.e. drug use, drug abuse or drug dependence) were higher in adulthood among ADHD cases compared with controls (27.7% vs. 18.1%, Table 3). Notably rates of drug *use* and *abuse* were similar while ADHD cases were approximately 3.5 times more likely to develop drug dependence (vs. no drug use) compared to controls (adjusted OR 3.48, 95% CI 1.38–8.79, p = 0.01). Simply put, as ADHD cases grew into adults, they were both more likely to use drugs and more likely to develop new onset drug dependence than controls. The effect is mediated through co-occurring childhood psychiatric diagnoses, which accounts for 39.2% of the association (p = 0.014).

**Table 3:** Current drug use, abuse and dependence among adults with versus without childhood ADHD, stratified by substance use disorder prior to 18 years of age.

Current Status	Adolescent Onset SUD = No			Adolescent Onset SUD = Yes		
	ADHD Cases(N = 177)	Non-ADHD(N = 320)	Adjusted OR‡(95% CI), P	ADHD Cases(N = 55)	Non-ADHD(N = 15)	Adjusted OR‡(95% CI), P
No drug use	128 (72.3%)	262 (81.9%)	Referent	28 (50.9%)	12 (80.0%)	Referent
Drug use withouta disorder	28 (15.8%)	38 (11.9%)	1.11 (0.63–1.98), 0.72	10 (18.2%)	1 (6.7%)	5.17 (0.56–47.86), 0.15
Drug abuse	6 (3.4%)	12 (3.8%)	0.81 (0.28–2.36), 0.69	7 (12.7%)	1 (6.7%)	2.97 (0.30–29.24), 0.35
Drug dependence	15 (8.5%)	8 (2.5%)	3.48 (1.38–8.79), 0.01	10 (18.2%)	1 (6.7%)	5.97 (0.64–55.61), 0.12

OR, odds ratio; CI, confidence interval.

† Drug (non-alcohol) substances include the following: stimulants, cocaine, narcotics, hallucinogens, inhalants, marijuana, and tranquilizers. In order to create mutually exclusive categories, each individual’s overall current non-alcohol substance use status was classified as the most severe status among all substances.

‡ Adjusted for age and gender.

*Adjusted for age.

Among the individuals who did have adolescent onset SUD, rates of drug exposure in adulthood tended to be higher in ADHD cases as compared to controls (49.1% vs. 20.0%; Table 3). However, given the limited sample size, we were unable to identify significant differences in the rates of non-alcohol drug use, drug abuse, or drug dependence in adulthood between ADHD cases and controls who had adolescent onset SUD.

### Alcohol Use in Adulthood by Adolescent Onset SUD Status

Among the subgroup of individuals with adolescent-onset SUD, those with ADHD were significantly more likely to have alcohol dependence (vs. no heavy alcohol use) as adults compared to controls (29.1% vs. 6.7%; adjusted OR 14.38, 95% CI 1.49–138.88, p = 0.02; Table 4). Our findings on alcohol dependence among the subgroup of adults who did not have a SUD in adolescence were distinctly different. In this group, ADHD cases were less likely to report heavy alcohol use (i.e. 3 or more drinks in a 3 hour period) (adjusted OR 0.56, 95% CI 0.36–0.88, p = 0.01) while there were no differences in the rates of alcohol abuse (adjusted OR 1.31, 95% CI 0.64–2.71, p = 0.46) or dependence (adjusted OR 0.94, 95% CI 0.47–1.89, p = 0.86).

**Table 4:** Current heavy alcohol use, abuse and dependence among adults with versus without childhood ADHD, stratified by substance use disorder prior to 18 years of age.

Current Status	Adolescent Onset SUD = No			Adolescent Onset SUD = Yes		
	ADHD Cases(N = 177)	Non-ADHD(N = 320)	Adjusted OR^‡^(95% CI), P	ADHD Cases(N = 55)	Non-ADHD(N = 15)	Adjusted OR^‡^(95% CI), P
No heavy alcohol use	77 (43.5%)	125 (39.1%)	Referent	14 (25.5%)	9 (60.0%)	Referent
Heavy alcohol use	60 (33.9%)	146 (45.6%)	0.56 (0.36–0.88), 0.01	20 (36.4%)	4 (26.7%)	3.12 (0.73–13.29), 0.12
Alcohol abuse	22 (12.4%)	21 (6.6%)	1.31 (0.64–2.71), 0.46	5 (9.1%)	1 (6.7%)	3.24 (0.29–36.65), 0.34
18 (10.2%)	18 (10.2%)	28 (8.8%)	0.94 (0.47–1.89), 0.86	16 (29.1%)	1 (6.7%)	14.38 (1.49–138.88), 0.02

OR, odds ratio; CI, confidence interval.

† Adjusted for age and gender.

*Adjusted for ag.

### Persistence of Alcohol and Non-Alcohol Substance Dependence from Adolescence to Adulthood

**Figure 1:**
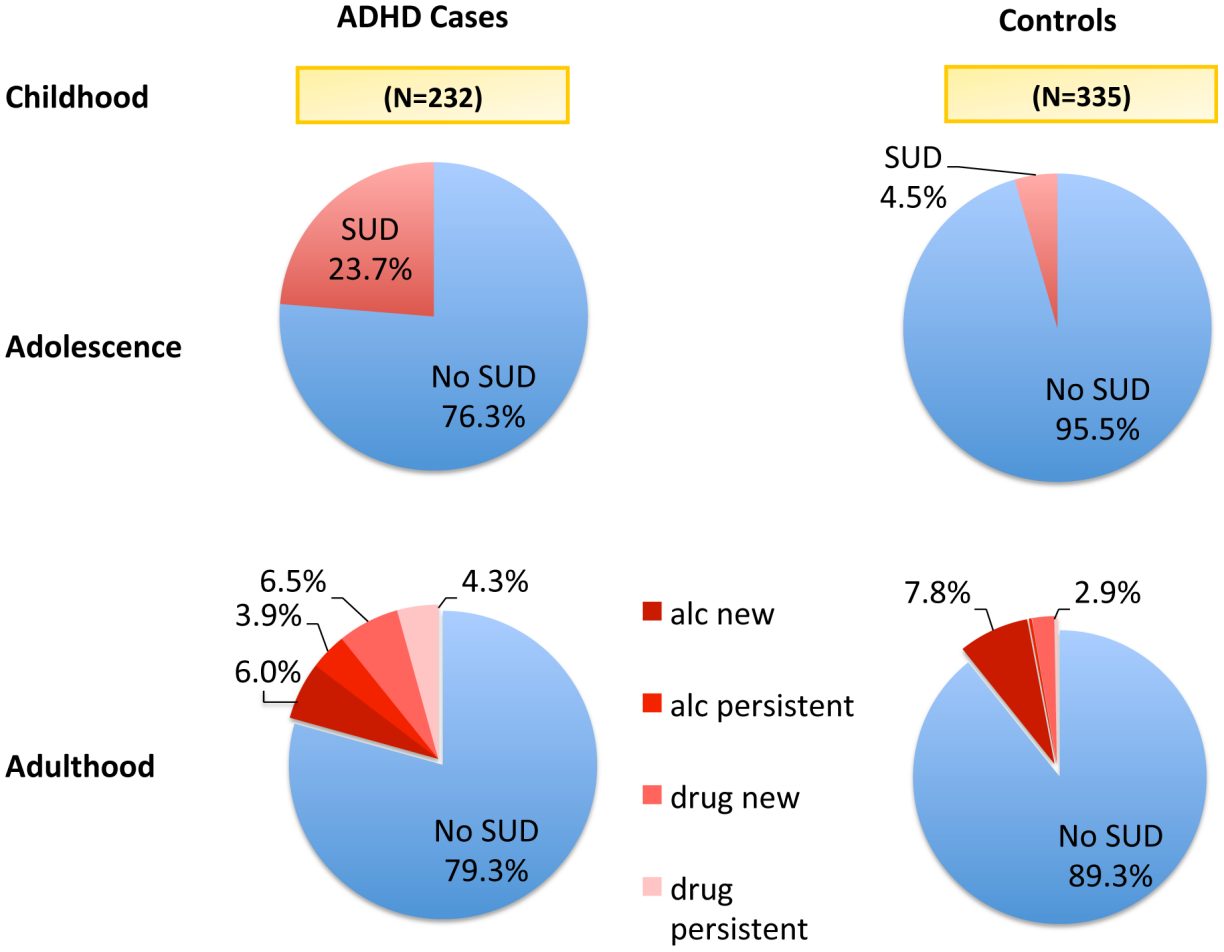
Adolescent SUD and adult substance dependence outcomes for childhood ADHD incident cases and controls. *Individuals with both “drug” dependence and alcohol dependence are coded as drug dependent in these figures. Individuals coded as alcohol dependent did not meet dependence criteria for any other substance.

The complex relationship between childhood ADHD, adolescent- onset SUD, and adult SUD’s are summarized in Figure 1. The overall rate of persistent SUD from adolescence into young adulthood was higher among childhood ADHD cases versus controls (19/232 or 8.2% versus 2/335 or 0.6%; p<0.001). Among the subgroup of subjects with adolescent-onset SUD, SUD persisted into adulthood for 34.6% for ADHD cases (19/55) versus 13.3% for controls (2/15). This finding was not statistically significant (p = 0.20) due to limited power, with only 15 controls having adolescent-onset SUD.

### Impact of Stimulant Treatment in childhood on Substance Dependence in Adulthood

Our previous, retrospective review of records found that 178 of the 232 ADHD cases received stimulant treatment in childhood, and information about the age at which stimulants were started and duration of treatment was available for 167. [23] Subjects who were treated for 6 months or longer (n = 139) were considered “treated” for analyses of the association between stimulant treatment in childhood and substance dependence in adulthood. For analyses of the association between stimulant treatment and risk for adult drug or alcohol dependence, we dichotomized subjects into those who received any treatment after the age of 13 years (n = 114) versus those who only received treatment prior to age 13 years (n = 25). We found no association between timing of stimulant treatment during childhood and classification as adult drug or alcohol dependence when each of these outcomes were analyzed separately; however, the combined risk for *either* adult drug or alcohol dependence was higher among subjects who had received at least some stimulant treatment after age 13 years (Table 5). Furthermore, there was no association between age of initiation, dosage, or duration of stimulant treatment and risk for SUD (data not shown).

**Table 5:** Impact of stimulant treatment in childhood on substance dependence in adulthood.

Substance Dependence Status	Stimulant treatment ended before age 13 (N = 25)	Stimulant treatment ended after age 13 (N = 114)	*P value*
Alcohol dependence	1 (4.0%)	19 (16.7%)	0.125
Substance dependence	1 (4.0%)	12 (10.5%)	0.463
Alcohol or substance dependence	1 (4.0%)	25 (21.9%)	0.046

### Impact of ADHD on treatment for substance dependence

We calculated the rates of substance dependence among ADHD cases and controls who had received any form of treatment for a SUD to examine whether ADHD moderated SUD treatment effects. Overall, we found that individuals with ADHD were more likely than controls to have received treatment for alcohol, marijuana, or other drug use disorders (26.7% vs. 9.3%; adjusted OR 3.37, 95% CI, 2.05–5.54, p<0.001). Forty-seven ADHD cases and 19 controls reported treatment for alcohol disorders. Among study participants who received treatment for an alcohol disorder, ADHD cases were more likely to have alcohol *dependence* in adulthood compared with controls (40.4% (19/47) vs. 15.6% (3/19); adjusted OR 3.84, 95% CI 0.95–15.57, p = 0.06). Few participants had received treatment for marijuana or another drug disorder, and we did not find statistically significant effects for other substances.

## Discussion

Our study provides initial evidence that individuals with childhood ADHD remain more susceptible than peers (without ADHD) to developing new onset *drug dependence,* a disorder associated with neurological changes in the brain, in adulthood. This finding suggests that early onset drug use, a factor known to increase the risk of developing substance dependence, does not fully account for the relationship between ADHD and SUD. Overall, the rate of drug exposure (i.e. drug use or drug abuse or drug dependence) was higher in adults with ADHD, suggesting that impulsivity and poor decision-making continue to play a role. However, among individuals who reported any drug use as adults, rates of new onset drug dependence was significantly higher among ADHD cases – suggesting that the relationship is multi-factorial and not fully explained simply by a greater propensity to use drugs. Furthermore, our findings suggest that individuals with ADHD are more likely than their non-ADHD peers to have persistence of substance dependence in young adulthood. Our data did not allow us to evaluate the relationship between individual drugs and adult onset substance dependence because of small cell sizes, and it is possible that results vary depending on substance. We note that marijuana was the most frequently used drug by adult participants in this study. It is important to note that “medical” marijuana was not recommended during the years when members of this birth cohort were children or adolescents, so these findings cannot be attributed to clinician recommendations. Most importantly, while some advocates for “medical” marijuana have suggested marijuana as a potential therapy for ADHD, our findings of heightened susceptibility to adult onset substance dependence give reason for pause.

The pre-frontal cortex is an area of the brain that has been implicated in ADHD, [26]–[28] and poor executive functioning is one of the core features of the disorder. [29]–[32] Perhaps not surprisingly, we found that adults with a history of childhood ADHD who did not have a SUD during adolescence were nevertheless significantly more likely than controls to develop drug dependence, a condition known to be associated with poor executive functioning during young adulthood. This finding is mediated by the presence of co-occurring mental health disorders, and we speculate that the maturation of executive functions might be further slowed in individuals with a mental health disorder, leaving them at very high risk for using drugs and developing drug dependence. This group should be counseled to avoid drugs and should be carefully monitored for SUDs beyond adolescence.

Our findings regarding alcohol use were different. Individuals with childhood ADHD and adolescent SUD were more likely to have *alcohol dependence* in adulthood. However, ADHD cases that did not develop a SUD in their teens were less likely than peers to drink heavily and they were *not* at increased risk for developing new onset *alcohol dependence.*

While all substances of abuse have action in the brain’s “reward center,” alcohol’s mechanism of action is distinct. Unlike marijuana and most other illicit substances, alcohol freely crosses cell membranes and exerts its actions on a variety of targets, rather than via a specific receptor. Alcohol dependence is highly heritable, with some individuals particularly prone to developing alcohol use disorders and others relatively resistant. For example, specific alleles for genes that encode alcohol dehydrogenase appear to be protective against alcoholism. Animal studies have found that the neuronal sensitivity to alcohol induced excitation is lower in mice that are bred to be “heavy drinkers.” [33].

We speculate that individuals with ADHD can be divided into two groups regarding liability for developing alcohol dependence. Individuals with a “high liability” develop alcohol use disorders in adolescence and have persistent alcohol dependence in adulthood. In other words, adolescents with ADHD may be less likely to “outgrow” an alcohol use disorder than their peers. As in reports from other authors, we found that treatment for SUD is less effective in this group. [34]–[39] On the other hand, individuals with ADHD who have a “lower liability” for alcohol problems may be *less* likely to drink heavily which may be protective from developing alcohol use disorders in both adolescence and adulthood. We cannot tell from our data whether this “liability” is inborn or present early in life, or the result of refraining from substance use during adolescence. Whatever the mediators, they seem to be specifically related to alcohol regulation and do not protect individuals from developing *drug dependence* as adults. While the causes remain unclear, it seems prudent to counsel adolescents with ADHD to avoid alcohol use. The National Institute on Alcohol Abuse and Alcoholism (NIAAA) has recommended alcohol screening and brief intervention for children as young as 9 years old, the age prior to initiation of alcohol use for most individuals. [40] Our findings highlight the importance of preventative alcohol counseling, particularly for individuals with ADHD.

In our previous retrospective study of the association between stimulant treatment and risk for SUD through adolescence in this cohort of childhood ADHD cases, we found that among boys who were treated, 21.8% had substance abuse compared to 36.4% for those not treated. [1] However, among girls who were treated (N = 10), 15.2% had substance abuse compared to 10.3% of those not treated, although the total number of girls with SUD in adolescence was small (n = 13). In the current, prospective study of SUD outcomes in adulthood, subjects who received stimulant treatment for at least 6 months, and who received some treatment after age 13 years, were at *greater* risk for adult drug or alcohol dependence than subjects who only received stimulant treatment prior to age 13 years. Given our finding of increased risk for SUD developing after adolescence among ADHD cases, it should not be surprising that stimulant treatment ending in mid-adolescence would be unlikely to continue to protect against the subsequent development of drug or alcohol dependence. Furthermore, although it cannot be determined from our dataset, subjects who continued to receive treatment after age 13 years may have had more severe ADHD symptoms and hence may have been at greater risk for development of SUD. Future studies that ensure continued stimulant treatment of ADHD through young adulthood may offer an opportunity to better understand the potential impact of stimulant treatment on risk for SUD into adulthood.

Our study has a number of strengths. Our data came from a large, population-based birth cohort and did not depend on a sample referred for ADHD treatment, which typically over represents individuals with the most challenging disorders. Our birth cohort was large, retrospective data was extensive, and our prospective data included a comprehensive, structured neuropsychiatric interview, making our data set unique among published studies of ADHD outcomes. We also note a number of limitations. Our retrospective data did not include age of initiation of substance use, which was not routinely noted in the medical record. Thus, we were unable to evaluate the relationship between age of initiation of substance use and outcomes. Retrospective data were not sufficiently detailed to distinguish between substance *abuse* and substance *dependence* during adolescence nor to determine which substances were used during adolescence. Our control group was well matched on demographic measures, though co-occurring mental health disorders were more common in ADHD cases than controls, and these disorders play a role in the relationship between ADHD and SUD. It is also possible that other, unmeasured factors may confound our findings. Although our initial cohort of ADHD cases was large, the number of subjects with adolescent-onset SUD followed into adulthood was smaller, contributing to relatively large confidence intervals around estimates of rates of adult SUD in this sub-group; hence, these specific results should be interpreted with caution.

## Conclusions

This report is the first to utilize a population-based birth-cohort to examine the relationship between childhood ADHD and SUD during young adulthood and to describe differential rates of new onset substance dependence in adults with a history of childhood ADHD. Our findings make an important contribution in understanding the intersection between these two common disorders. Most importantly, our findings underscore the importance of continuous monitoring, prevention and early intervention for substance use among children, adolescents, and adults with ADHD to prevent the development of substance dependence. Further study is needed to determine the most effective mechanisms for achieving these goals.
